# Ant mediated redistribution of a xyloglucanase enzyme in fungus gardens of *Acromyrmex echinatior*

**DOI:** 10.1186/s12866-016-0697-4

**Published:** 2016-05-06

**Authors:** Pepijn W. Kooij, Jeroen W. M. Pullens, Jacobus J. Boomsma, Morten Schiøtt

**Affiliations:** Centre for Social Evolution, Department of Biology, Copenhagen University, Universitetsparken 15, Copenhagen, DK-2100 Denmark; Laboratory of Genetics, Wageningen University and Research Centre, P.O. Box 309, Wageningen, 6700 AH The Netherlands; Present address: Jodrell Laboratory, Royal Botanic Gardens, Richmond, Kew TW9 3DS UK

**Keywords:** Leaf-cutting ants, *Leucoagaricus gongylophorus*, Gene expression, Stoichiometry, Xyloglucan, Plant cell wall degradation, Mutualism

## Abstract

**Background:**

Xyloglucan is an important component in plant cell walls that herbivores cannot digest without microbial symbionts. Leaf-cutting ants are major insect herbivores in the Neo-Tropics that rely on fungus-garden enzymes for degrading plant cell walls. However, many of these ants discard much of their harvested plant material after partial degradation, which has led to the hypothesis that the fungal symbionts are primarily producing cell wall degrading enzymes to gain access to intracellular nutrients rather than for obtaining sugars from recalcitrant cell wall polymers, such as (hemi-)cellulose.

**Results:**

The fungal symbiont provides a single xyloglucanase (Xeg1) to its ant farmers by upregulating the expression of this protein in the inflated hyphal tips (gongylidia) that the ants ingest. Similar to other enzymes ingested this way, also Xeg1 is not digested but vectored to the fresh leaf-fragment pulp at the top of fungus gardens via ant fecal fluid. Xeg1 is 4-5 times more active in fecal fluid when ants ingest their normal fungal food, compared to a sucrose control diet, as expected when they cannot produce Xeg1 themselves. We confirm substrate specificity of fungal Xeg1 towards xyloglucan by heterologous expression in yeast and show that xyloglucanase activity is higher in the oldest, bottom layers of fungus gardens and in discarded debris material than in the upper and middle layers of fungus gardens.

**Conclusion:**

Our results are consistent with Xeg1 playing a role in the initial breakdown of plant cell wall hemicellulose to provide sugars for aggressive hyphal growth before intracellular proteins become available. Xeg1 does not play a major decomposition role in the middle layer of fungus gardens where it is produced by the gongylidia. Overall high xyloglucanase activity in old mycelium that is (about to be) discarded is striking and quite possibly serves defensive purposes by precluding that competing microorganisms can grow. Our results support the hypothesis that the ant-fungus symbiosis prioritizes access to the protein-rich contents of live plant cells and that carbohydrates are not a limiting resource.

**Electronic supplementary material:**

The online version of this article (doi:10.1186/s12866-016-0697-4) contains supplementary material, which is available to authorized users.

## Background

Plant cell wall polymers are among the most abundant natural resources, but very few animals have genomes that encode enzymes with the capacity to degrade these compounds [1]. Most animals that specialize on plant-based diets, therefore, live in symbioses with microorganisms that produce these enzymes for them. Symbioses of this kind are not only common in ungulates, but also in insect lineages where feeding on dead wood, leaf litter or live plant material has evolved multiple times independently [2, 3]. The gut symbionts involved are usually bacteria, but protists fulfill similar roles in the lower wood-dwelling termites [4, 5] and similar services can be provided by fungi, such as gut yeasts in several families of wood-ingesting beetles [6, 7], ambrosia fungi in wood-boring Xyleborine beetles [8], and *Termitomyces* fungi in fungus-growing termites [9].

The lower attine fungus-growing ants use dead plant material to manure their fungus-gardens, which implies that these fungi must have considerable plant cell wall degrading capacities [10]. However, in contrast to other insect-fungus symbioses, the attine ants evolved an evolutionarily derived leaf-cutting ant lineage that relies on active herbivory. After resolving challenges of defensive secondary plant compounds [11], this advanced symbiosis gained access to vast amounts of intracellular carbohydrates, lipids and proteins that would no longer be available in dead plant cells. However, although the fungi cultivated by leaf-cutting ants have retained the capacity to degrade all major components of plant cell walls except lignin [12–20], many leaf-cutting ants discard large amounts of non-decomposed plant cell wall material from the bottoms of their fungus gardens [14, 17], suggesting that further digestion of this material is not of high priority. It has therefore been suggested that the main purpose of fungal symbiont cell wall degrading enzymes is to macerate the fresh plant tissues deposited on the top of gardens merely to gain access to more easily digestible intracellular nutrients [13, 21–23].

The two genera of leaf-cutting ants, *Atta* and *Acromyrmex*, live in obligate mutualistic symbiosis with varieties of *Leucoagaricus gongylophorus* [24–26]. This highly productive polyploid crop-symbiont [26] has likely been instrumental for the ants’ progression to dominant herbivore status in the New World tropics [18, 27] with significant accelerator functions of nitrogen and phosphorus recycling in many ecosystems [28]. The phylogenetically basal “lower” attine ants rear saprotrophic fungus gardens, but the crops of leaf-cutting ants belong to a uniquely domesticated lineage that has evolved special symbiotic organs, staphylae, that grow bundles of inflated hyphal tips (gongylidia) to provide essentially all nutrition for the ants and their brood [29–31]. After the larger foraging workers have cut the leaf fragments and transported them back to the nest, smaller workers chew these pieces into a pulp, reducing the crystallinity of the plant cellulose [20, 32], and place this material on top of the fungus garden before manuring it with fecal fluid and inoculating it with fresh tufts of mycelium [13].

The ant fecal fluid is involved in the rapid maceration of leaf pulp [22], and contains a wide range of fungal plant degrading enzymes such as pectinases [13], laccases [11] and proteases [33]. The genes coding for these enzymes are almost always upregulated in the gongylidia that the ants ingest and the enzymes themselves normally retain their activity after gut passage so they can be vectored to garden locations where they are most useful by the ant workers. However, gongylidia upregulation and fecal fluid expression for cellulases and hemicellulases has not been investigated previously, as these categories of enzymes were not particularly common among the ca. 36 proteins identified in the *Acromyrmex echinatior* fecal fluid [13]. In fact, only a single protein (Xeg1) belonging to the glycoside hydrolase family 12 was identified in the fecal fluid of Panamanian *A. echinatior* [13], but remained unconfirmed in a recent study on lignocellolytic enzymes in *L. gongylophorus* from other sympatric colonies of the same ant species [16].

Substrates targeted by the members of the GH12 enzyme family are cellulose, xyloglucan and β-1,3-1,4-glucan. Cellulose is the dominant component of both primary and secondary plant cell walls, whereas xyloglucan is the most abundant hemicellulose in the primary cell wall of dicotyledons, comprising ca. 20 % of the plant cell wall dry weight [34] and having important roles in plant cell wall defenses [35]. We therefore hypothesized that the identified GH12 enzyme might have an important function in fecal fluid activity, and designed the present study to: 1. Measure gene expression levels of *xeg1* in the different layers of the fungus garden and in the fungal gongylidia relative to normal mycelium, 2. Identify the substrate specificity of Xeg1, 3. Confirm that Xeg1 activity in the fecal fluid is fungal derived, 4. Measure the xyloglucanase activity in both fecal fluid and fungus gardens of *Acromyrmex echinatior*, and 5. Discuss the implications of our findings for the ongoing discussion about the functional priorities of plant cell wall degradation by leaf-cutting ant fungus gardens.

## Methods

### Biological material

A total of seven ant colonies of *Acromyrmex echinatior* (Ae226, Ae263, Ae266, Ae280, Ae322, Ae335 and Ae370), collected between 2004 and 2007 in Gamboa, Panama, were used for this study. All colonies were kept under controlled conditions of ca. 25 °C and a relative humidity of ca. 70 %, and were fed twice a week with fresh bramble leaves (*Rubus sp.*), parboiled rice and pieces of apple, which they use to maintain their fungal symbiont, *Leucoagaricus gongylophorus*.

For the collection of fecal fluid, the gaster (final abdominal segments) of a large worker was gently pressed onto a glass microscope slide using a forceps [36]. Each fecal droplet was mixed with 0.5 μl demineralized and sterilized water (the fecal fluid “enzyme extract”) and collected with a micropipette in an Eppendorf vial and kept on ice until further processing. Fecal fluid was collected from two different groups of large workers for each of five colonies (Ae226, Ae263, Ae266, Ae280 and Ae322), one group having access to the fungus garden and thus assumed to have ingested fungal gongylidia, and the other being deprived of their fungus garden and provided only with 10 % sucrose water for 20 days. We further collected 120 mg fungus garden material from each of the three visible layers of the source colony fungus garden [15], the fresh and dark top layer, the mature gongylidia-producing middle layer [11] and the old bottom layer from five colonies (Ae226, Ae263, Ae266, Ae280 and Ae322). We supplemented this material with samples from the colony’s debris material, i.e. old fungus garden material discarded by the ants, similar to a previous study [15] and added 500 μl of demineralized and sterilized water to each sample and crushed material with a pestle before vortexing and centrifuging for 15 min (15,000 g). The fungus garden supernatant and the fecal fluid extract were then kept on ice until processing later on the same day.

For gene expression measurements in gongylidia and control mycelium, we collected garden material from five colonies (Ae263, Ae280, Ae322, Ae335 and Ae370): ca. 100 μl of gongylidia clusters (staphylae) and a similar mass of normal mycelium in separate 2 ml Eppendorf tubes floating in liquid nitrogen, using a stereomicroscope at 40x magnification. For gene expression measurements in the different fungus garden layers and the debris pile, these samples were supplemented by 120 mg fungal material from colonies Ae226, Ae263, Ae266, Ae280 and Ae322, after carefully removing any visible ants or ant parts, larvae and eggs. All samples were stored at -80 °C for RNA extraction at a later point.

### Protein identification and gene cloning

SDS-PAGE and mass spectrometry was performed as described previously [11, 13, 33]. A mix of fifty fecal droplets was subjected to SDS-PAGE, after which individual protein bands were manually excised from the gel. Proteins were extracted from the gel plugs and digested with trypsin. De novo sequencing of the resulting peptide fragments was performed on the basis of b and y fragment ions present in MS/MS spectra of derivatized and underivatized samples. Amino acid sequences were obtained by manual analysis of the spectra using the software program AminoCalc (Protana A/S, Odense, Denmark) as support. The obtained amino acid sequences were used as queries in a Blast search of the assembled *Acromyrmex echinatior* genome [37] and a low coverage genome sequence of the *Acromyrmex echinatior* fungal symbiont [11].

RNA extraction and cDNA production was performed as described in Schiøtt et al. [13]. A full-length gene transcript sequence of *xeg1* was obtained using a RACE (Rapid Amplification of cDNA Ends) strategy. 3’ and 5’-RACE libraries were made from ca. 1 μg of extracted RNA with the SMART RACE cDNA kit (CLONTECH, Mountain View, California, USA), and 3’end and 5’end gene sequences were PCR amplified from these libraries using the gene specific primers SYT-F1 (CAG TCG ACA CTT CCC AGC ACT GTG) or SYT-R1 (GGT ATA CTG ACC AGC AGT GAC GGT G) designed from the BLAST-search-identified sequence reads along with the UPM primer enclosed in the SMART RACE cDNA kit. The following PCR scheme was used in the RACE experiments: one cycle of 95 °C for 5 min, 10 cycles of 95 °C for 20 sec, 72 °C for 30 sec (with a decrease in temperature of 0.5 °C in every cycle) and 72 °C for 3 min, followed by 35 cycles of 94 °C for 20 sec, 67 °C for 30 sec and 72 °C for 3 min, and ending with one cycle of 72 °C for 7 min. PCR products were cloned in pCR4-TOPO using the TOPO-TA cloning procedure (Invitrogen, Carlsbad, California, USA) and sequenced at Eurofins MWG Operon (Ebersberg, Germany). The full length gene sequence is deposited in GenBank with accession number KU198622.

### Quantitative real-time PCR

Primers for the *xeg1* gene (Additional file 1: Table S1) were designed by matching the obtained cDNA sequences to the available genome sequence of the *Acromyrmex echinatior* symbiont [11] using BLASTn. Matching reads were assembled and aligned to the cDNA sequence to identify intron and exon sequences. Primers were then designed to span an intron to avoid amplification of genomic DNA. The qPCRs were run on an Mx3000P QPCR System (Agilent, Santa Clara, CA, USA) in a 20 μl reaction (0.5 μl cDNA, 10 μl 2x SYBR Premix Ex Taq [TaKaRa Bio Inc., Otsu, Japan] and 0.4 μl of each primer [10 μM]) with the following conditions: one cycle of 95 °C denaturing for 2 min, followed by 40 cycles of 95 °C denaturing for 30 sec, 57 °C annealing for 30 sec, and 72 °C extension for 30 sec and ending with a melting curve cycle of 95 °C for 30 sec, 57 °C for 30 sec and 95 °C for 30 sec.

We performed qPCR for three genes in total: the target gene *xeg1* and two housekeeping genes *ubiquitin* (*Ubc*, GenBank HQ174771) and *glyceraldehyde 3-phosphate dehydrogenase* (*GAPDH*, GenBank HQ174770) with three technical replicates for each sample. All Ct values from the RT-qPCR analyses were analysed using R with packages “ReadqPCR” [38] and “NormqPCR” [39]. Average expression was calculated from the three technical replicates of each sample for two datasets, one with Ct values for the different fungus garden layers including the debris pile and the other with Ct values for the fungus garden mycelium and gongylidia.

Based on the primer efficiencies for all genes (*xeg1*: 95.6 %; *Ubc*: 95.8 %; *GAPDH*: 91.6 %), only *Ubc* was used as reference housekeeping gene to determine the normalized expression values for *xeg1* in the fungus garden layer samples, which produced 2^∆Ct^ values [40] for each layer. These results were further analysed with R using ANOVA to generate individual p-values with General Linear Hypotheses (“glht”) in the Simultaneous Inference in General Parametric Models package “multcomp” [41]. *Ubc* was also used as housekeeping gene for the gene expression comparison between gongylidia and mycelium, to calculate the relative expression levels of the *xeg1* gene, resulting in 2^-∆∆Ct^ values [42].

### Heterologous expression in yeast

Heterologous expression of the cDNA sequence of *xeg1* in *Saccharomyces cerevisiae* was done to identify the enzymatic activity of the encoded protein. The coding sequence of the *xeg1* gene was PCR amplified from cDNA using the forward GH12-F1 primer 5’-AAG CTT AAA TAA TGT TCT CCA AGA AAT TGA CTG C-3’ together with the reverse GH12-R1 primer 5’-TCT AGA CTA AGC GAT TGT CAC ACT ATA C-3’. PCR conditions were one cycle of 94 °C for 5 min, then 30 cycles of 94 °C for 30 sec, 55 °C for 30 sec and 72 °C for 1 min, and ending with one cycle of 72 °C for 10 min. To increase the product, the amplified cDNA was cloned in pCR4-TOPO using the TOPO TA cloning method (Invitrogen, Carlsbad, CA, USA), after which the clone was sequenced to ensure that no PCR errors had been incorporated into the construct. Through digestion and ligation steps the construct was then inserted into the yeast expression vector pYES2 (Invitrogen) under he control of a galactose inducible promoter. *Saccharomyces cerevisiae* strain INVSc1 (MATa his3D1 leu2 trp1-289 ura3-52, Invitrogen) was transformed with either the pYES2 vector containing the *xeg1* gene (pYES2-*xeg1*) or an empty pYES2 vector using the LiOAc/polyethylene glycol (PEG) method [43]. As the pYES2 vector contains the *URA3* gene, positive transformants could be selected by plating on a uracil depleted yeast medium consisting of 2 % glucose, 2 % bacto-agar, 0.7 % (wt/vol) yeast nitrogen base (Invitrogen) and 0.2 % yeast synthetic drop-out media supplement without uracil (Sigma- Aldrich), and incubating at 25 °C for 7 days. Ura^+^ colonies were then grown in a similar medium without agar and with either 2 % glucose or 2 % galactose at 25 °C overnight. Yeast cells from 5 ml cultures were harvested by centrifugation, ground in liquid nitrogen with a mortar and pestle, and dissolved in an equal amount of 50 mM Tris pH 7.0 to be used in an AZCL assay for four different substrates; barley β-glucan (endo-β-1,3-1,4-glucanase), HE-cellulose (endo-β-1,4-glucanase), pachyman (endo-β-1,3-glucanase) and xyloglucan (endo-β-1,4-xyloglucanase).

### Xyloglucanase activity

Fecal fluid and fungus garden xyloglucanase activities were measured using the AZO-Xyloglucan-assay (Megazyme, Wicklow, Ireland). Xyloglucanase activity was determined by incubating 50 μl enzyme extract in 100 μl pre-equilibrated substrate solution, pH 5. The solution was vortexed and incubated for 10 minutes at 30 °C and the reaction terminated by adding 250 μl of 99 % ethanol. Samples were then vortexed and centrifuged for 10 minutes at 1000 g, after which the supernatant was measured for absorbance at 590 nm in the Versamax ELISA micro plate reader (Molecular Devices, Sunnyvale, California, USA). The absorbance measurements were converted to enzyme units using the standard curve included in the protocol. One unit was defined as the amount of enzyme required to release 1 μg of glucose per minute per fecal droplet or mg fungus garden.

## Results

Mass spectrometry analysis of fecal fluid from *Acromyrmex echinatior* [11, 13, 33] identified the peptide sequence SYTNJNJNAN(LNK) (in band number 12 of the SDS-PAGE picture presented in an earlier publication [13]). The J can be either leucin or isoleucin which have the same mass, and the identities of the last three amino acids could not be unequivocally determined by mass spectrometry, but had a cumulated mass that fits with LNK and matched the *xeg1* gene in the genome of *Leucoagaricus gongylophorus* [11, 17] and the corresponding amino acid sequence SYTNINLNANLNK. BLAST search allowed us to assign the *xeg1* gene to glycoside hydrolase family 12. Relative changes in *xeg1* gene expression in gongylidia versus normal mycelium (fold-changes: 2^-∆∆Ct^) showed that the gene was significantly more expressed (fold change = 3.6, SE-min – SE-max = 0.48 – 5.30) in the gongylidia than in normal mycelium (z = -5.339, *p* < 0.00001; Additional file 1: Table S2), indicating that ingestion of this enzyme by the ants for transfer to the fecal fluid is actively promoted by the fungal symbiont. We found no differences in normalized gene expression of the *xeg1* gene across the different fungus garden layers (F_3,16_ = 1.988, *p* = 0.156; mean ± SE normalized fold changes per layer: top: 0.572 ± 0.105; middle: 0.652 ± 0.411; bottom: 0.359 ± 0.067; debris material: 1.671 ± 0.710; Additional file 1: Table S3).

**Fig. 1:**
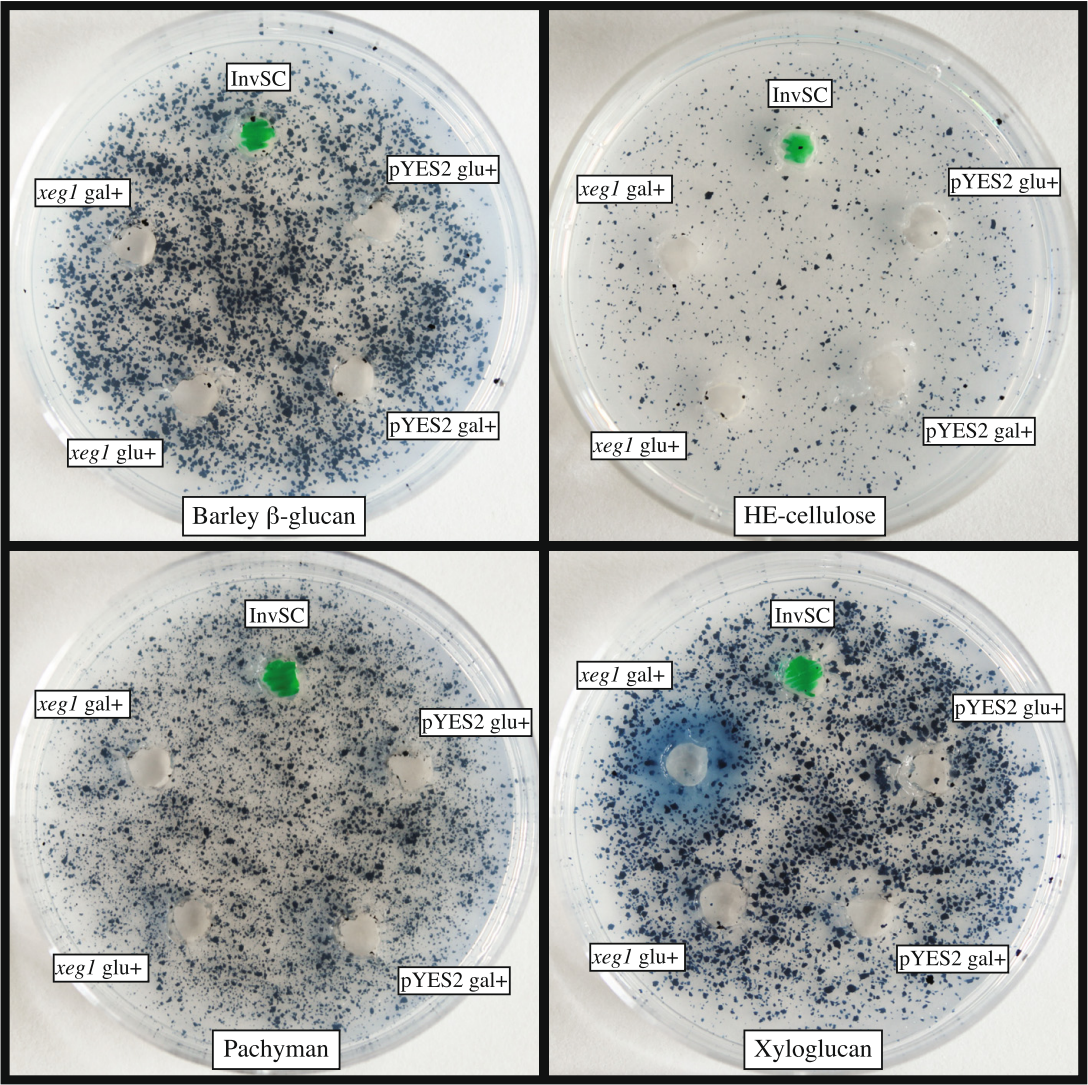
Heterologous expression of *xeg1* in yeast shows specificity towards xyloglucan. Enzyme activity on barley β-glucan, HE-cellulose, pachyman and xyloglucan after heterologous gene expression of *xeg1* from fungus gardens of *Acromyrmex echinatior* in yeast. Tested samples are, from the top and clock-wise, empty InvSC (marked green to simplify the identification of samples after incubation), InvSC-pYES2 from glucose medium, InvSC-pYES2 from galactose medium, InvSC-pYES2-*xeg1* from glucose medium and InvSC-pYES2-*xeg1* from galactose medium. No activity could be detected for any of the samples except for the activated (gal+) InvSC-pYES2-*xeg1*, which confirmed that Xeg1 is a xyloglucanase

The *xeg1* gene was successfully inserted into yeast using the pYES2 vector with a galactose inducible GAL1 promoter, and was subsequently found to be active only for the activated (gal+) InvSC-pYES2-*xeg1* transformant on xyloglucan whereas no activity was found for the other substrates, showing that Xeg1 is indeed a xyloglucanase (Fig. 1). Furthermore, the four controls, empty InvSC, InvSC-pYES2 in glucose medium, InvSC-pYES2 in galactose medium and InvSC-pYES2-*xeg1* in glucose medium showed no activity, proving that the activity of the activated (gal+) InvSC-pYES2-*xeg1* transformant is indeed due to the activation of the *xeg1* gene.

**Fig. 2:**
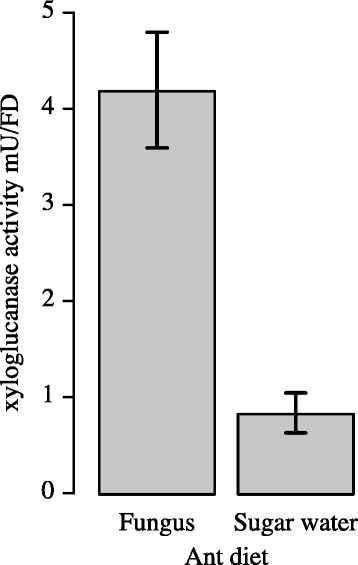
Xeg1 acitivity in fecal fluid is 4-5 times higher when ants eat fungus garden material. Xyloglucanase activities for fecal fluid produced by large workers of *Acromyrmex echinatior* provided with either a normal fungus garden diet or with sucrose water for 20 days. A Welch two sample t-test showed a significant reduction in xyloglucanase activity (t_4.943_ = 5.276, *p* < 0.01) when ants were deprived of fungus garden material. Enzyme activity levels are given as milliunits per fecal droplet (mean ± SE, *n* = 5 colonies)

**Fig. 3:**
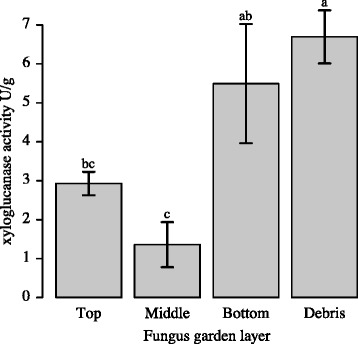
Xyloglucanase activity is highest in the bottom and discarded layers of fungus gardens. Xyloglucanase activities for the top, middle and bottom layers of *Acromyrmex echinatior* fungus gardens and debris material. Xyloglucanase activity was relatively low in the top and middle layers and high in the bottom layer and debris material (F_3,12_ = 7.246, *p* < 0.01). Enzyme activity levels are given as units per gram fungus garden material (mean ± SE, *n* = 5 colonies). Different letters above bars refer to differences in post-hoc tests at *P* < 0.05

In order to correlate the *xeg1* gene expression measurements in the gongylidia and the fungus garden layers with actual enzyme activity, we measured the xyloglucanase activity in the ant fecal fluid and in the same fungus garden layers that were used for the gene expression measurements. Fecal droplet xyloglucanase activity assays (Fig. 2) showed a substantial reduction in activity when ants had been feeding on sucrose water for 14 days (t_4.943_ = 5.276, *p* < 0.01; Additional file 1: Table S4), but some activity remained and was significantly higher than zero (t_4_ = 4.018, *p* < 0.01), suggesting that gut bacteria may also produce some xyloglucanase. The xyloglucanase activities in the different fungus garden layers increased from top to bottom/debris material (F_3,12_ = 7.246, *p* < 0.01, Fig. 3; Additional file 1: Table S5), with activity being slightly higher in the top compared to the middle layer, consistent with fecal droplets being placed at the top of the fungus garden. However, the highest activities were found in the bottom layer of the fungus garden and the debris material, consistent with previous findings observing a drop in the amount of xyloglucan present in fungus gardens from middle to bottom layer and from bottom layer to debris material [14].

## Discussion

Breaking down live plant cell walls is a complex process, which involves multiple groups of enzymes to degrade this matrix of cellulose fibers intertwined with pectins, lignins and hemicelluloses. The enzymes normally involved are: 1. polyphenol oxidases (including laccases), which break down lignin and secondary metabolites that function as defensive compounds, 2. pectinases and hemicellulases, which open-up the matrix of the cellulose fibers, 3. proteases, which degrade cell wall proteins, and 4. cellulases that break down the long cellulose chains into single glucose molecules [34, 44]. So far, it has been shown that a special group consisting of one laccase [11], six pectinases [13] and five proteases [33] are all produced in enhanced quantities in the gongylidia of leaf-cutting ant fungal cultivars. These enzymes are now also known to pass undigested through the gut system of the ants to end up in the fecal fluid, which the ants mix with their newly collected leaf pulp when adding this to the top of fungus garden. Our present study shows that a similar scenario holds for the xyloglucanase function of the hemicellulase Xeg1, which completes a survey of tracing down all abundant enzymes isolated from the fecal droplets of *A. echinatior* [13] and without having found a single enzyme that targets recalcitrant cellulose or lignin.

Several recent studies have confirmed that pectinases in particular have important roles in the pre-digestion of plant material in the top layers of fungus gardens while cellulose remains mostly undigested until these residues have reached the bottom layer and the debris material [14, 16, 17, 22, 23, 45]. Our results show that also xyloglucanases have a higher activity towards the bottom layer of the fungus garden and in the debris material (Fig. 3), complementing an earlier study showing a similar stratification for xylanases [23]. These results imply that the fungus garden can indeed convert xyloglucan into glucose molecules that can be taken up as nutrients by fungal cells, but that this activity is mostly expressed towards the end of the substrate degradation process when other more easily accessible nutrients have already been decomposed. Although many studies have pointed out that high (hemi-)cellulolytic activity in the bottom of fungus gardens may also be due to bacteria [45–47] or yeasts [48–50], the present study and recent results by Aylward et al. [16] and Grell et al. [17] are consistent in suggesting that a significant fraction of (hemi-)cellulose decomposition in the bottom of gardens is performed by the fungal symbiont itself. Our results also show that hemicellulase activity continues to be high in the bottom layer and debris pile, which suggests that fungal symbiont cells survive in this untended environment in substantial quantities. These activities are unlikely to benefit the ant colony directly as the bottom of fungus gardens hardly produces gongylidia [11], but they make sense from a hygienic perspective as it limits niche space for alternative decomposers that might spread as infections to the bottom of fungus gardens when allowed to get a foothold in the waste piles [17].

In addition to confirming hemi-cellulolytic activity towards the end of the decomposition process, our present study also shows that xyloglucan decomposition is an explicit target in the top of fungus gardens. An even more pronounced gongylidial upregulation of the *xeg1* gene (ca. 20 fold) was found in a recent RNA-seq study of leaf-cutting ant fungal symbionts, where this gene showed the largest difference in expression between gongylidia and mycelium among all genes predicted in the transcriptome [51]. This is an interesting result because, even though the GH12 enzyme family includes both xyloglucanases and cellulases, the Xeg1 enzyme specifically catabolizes xyloglucan but not cellulose (Fig. 1), consistent with the hypothesized purpose of just loosening up the plant cell walls to allow hyphal access to the cell interior with proteins and starch [13, 14, 17, 33]. At the same time, the sucrose released by early xyloglucan decomposition undoubtedly stimulates hyphal growth in the top of gardens before the hyphae have access to the starch inside the plant cell walls. Taken together these layer-specific enzyme activities confirm that cellulose degradation is not a priority and that decomposition is primarily focused on obtaining nitrogen and possibly other limiting nutrients, but not carbon.

This interpretation of our results follows the general idea that decomposition of organic matter takes place in a stoichiometric manner, which keeps ratios of liberated carbon, nitrogen, phosphorus and other possibly limiting nutrients relatively constant and releases any surplus non-limiting nutrients into the external environment [52–54]. It is well documented that plant diets offer an excess of carbon relative to nitrogen and phosphorus [52, 55], so that decomposers such as the ant-fungus symbiosis should be under selection to primarily invest in enzymes for acquiring the most limiting nutrients (nitrogen and possibly phosphorus), while adjusting carbon decomposition/liberation to the level needed for achieving stoichiometric equilibrium. Indications of the leaf-cutting ant symbiosis to be nitrogen-limited have emanated from the demonstration of nitrogen-fixing bacteria in the fungus gardens of *Atta* leaf-cutting ants [56] and other nitrogen conserving bacteria in the guts of *Acromyrmex* leaf-cutting ants [57]. Depending on the efficiency of these nitrogen preserving bacterial symbionts, it may thus turn out that the availability of phosphorus in the plant substrate is ultimately the most limiting factor for the leaf-cutting ant symbiosis, but further work will be needed to substantiate this hypotheses.

## Conclusion

The fungal symbiont of the leaf-cutting ant *A. echinatior* provides a single xyloglucanase (Xeg1) to the farming ants by upregulating the expression of this protein in the gongylidia that the ants ingest. Xeg1 is not used by the ants for digestion but for deposition on new leaf-fragment substrate via their fecal fluid, where it contributes to the degradation of recalcitrant plant cell wall polymers. Our results support the idea that the availability of carbon in the form of monosaccharides is not a limiting factor in the ant-fungus-farming symbiosis, which more likely is limited by other nutrients such as nitrogen and phosphorus, and the function of the fecal droplet xyloglucanase is to contribute to the breakdown of plant substrate cell walls to allow the fungus to gain access to the more nutritious intracellular compounds.

## Ethics and consent to participate

Permission to collect ant colonies and export them from Panama to Denmark was given by the Autoridad Nacional del Ambiente y el Mar (ANAM), Panama.

## Consent to publish

Not applicable.

## Availability of data and materials

All the data supporting the findings of this study are contained within the manuscript and its additional file.

## Additional file

Additional file 1: Table S1. List of qPCR primers used in this study, including primer sequences, annealing temperatures and fragment lengths in base pairs. **Table S2.** qPCR results of *Xeg1*, *Ubc* and *GAPDH*, for the two different fungal tissues, gongylidia and mycelium. **Table S3.** qPCR results of *Xeg1*, *Ubc* and *GAPDH*, for the top, middle and bottom layers and debris material from ant fungus gardens. **Table S4.** Xyloglucanase enzyme activity data for ant fecal fluid. **Table S5.** Xyloglucanase enzyme activity data for the top, middle and bottom layers and debris material from ant fungus gardens. (XLSX 28 kb)

## Abbreviations

*A. echinatior*: *Acromyrmex echinatior*
ANOVA: Analysis of Variance
AZCL: Azurine-Crosslinked
BLAST: Basic Local Alignment Search Tool
cDNA: Complementary Deoxyribonucleic Acid
DNA: Deoxyribonucleic Acid
ELISA micro plate reader: Enzyme-linked Immunosorbent Assay micro plate reader
GAL1: Galactokinase
GAPDH: Glyceraldehyde 3-Phosphate Dehydrogenase
GH12: Glycoside Hydrolase Family 12
glht: General Linear Hypotheses
INVSc1: *Saccharomyces cerevisiae* Yeast strain used in this study
LiOAc: Lithium Acetate
MS/MS: Tandem Mass Spectrometry
PCR: Polymerase Chain Reaction
PEG: Polyethylene Glycol
pYES2: Yeast expression vector used in this study
qPCR: Quantitative Polymerase Chain Reaction
RNA: Ribonucleic Acid
RT-qPCR: Real Time Quantitative Polymerase Chain Reaction
SDS-PAGE: Sodium Dodecyl Sulfate Polyacrylamide Gel Electrophoresis
SE: Standard Error
SMART RACE: Switching Mechanism At the 5’-end of RNA Transcript Rapid Amplification of cDNA Ends
Tris: Tris(hydroxymethyl)aminomethane
Ubc: Ubiquitin
UPM: Universal Primer Mix
URA3: Orotidine 5’-phosphate decarboxylase
Xeg1: Xyloglucanase described in this study
